# Myth or fact: 3D-printed off-the-shelf prosthesis is superior to titanium mesh cage in anterior cervical corpectomy and fusion?

**DOI:** 10.1186/s12891-024-07213-7

**Published:** 2024-01-26

**Authors:** Haoyu He, Lei Fan, Guohua Lü, Xinyi Li, Yunchao Li, Ou Zhang, Zejun Chen, Hui Yuan, Changyu Pan, Xiaoxiao Wang, Lei Kuang

**Affiliations:** 1 Department of Spinal Surgery, The Second Xiangya Hospital of Central South University, Changsha, Hunan Province China; 2 Department of Spinal Surgery, Third Hospital of Changsha, Changsha, Hunan Province China; 3 Department of Medical Education, California University of Science and Medicine, Colton, CA USA

**Keywords:** Cervical spondylotic myelopathy, Anterior cervical corpectomy and fusion, 3D-printed prosthesis, Subsidence, Cost-effectiveness

## Abstract

**Background:**

To find out if three-dimensional printing (3DP) off-the-shelf (OTS) prosthesis is superior to titanium mesh cages in anterior cervical corpectomy and fusion (ACCF) when treating single-segment degenerative cervical spondylotic myelopathy (DCSM).

**Methods:**

DCSM patients underwent ACCF from January 2016 to January 2019 in a single center were included. Patients were divided into the 3DP group (28) and the TMC group (23). The hospital stays, operation time, intraoperative blood loss, and the cost of hospitalization were compared. The Japanese Orthopedic Association (JOA) scores and Neck Disability Index (NDI) were recorded pre-operatively, 1 day, 3, 6, 12, and 24 months post-operatively. Radiological data was measured to evaluate fusion, subsidence, and cervical lordosis. Patients were sent with SF-36 to assess their health-related quality of life (HRQoL).

**Results:**

The differences in operative time, intraoperative blood loss, and hospital stay were not statistically significant between groups (*p* > 0.05). Postoperative dysphagia occurred in 2 cases in the 3DP group and 3 cases in the TMC group, which all relieved one week later. The difference in improvement of JOA and NDI between the two groups was not statistically significant (*p* > 0.05). No hardware failure was found and bony fusion was achieved in all cases except one in the 3DP group. The difference in cervical lordosis (CL), fused segmental angle (FSA), mean vertebral height (MVH), and subsidence rates between groups at each follow-up time point was not statistically significant and the results of the SF-36 were similar (*p* > 0.05). The total cost was higher in the 3DP group with its higher graft cost (*p* < 0.05).

**Conclusion:**

In treating single-segment DCSM with ACCF, both 3DP OTS prosthesis and TMC achieved satisfactory outcomes. However, the more costly 3DP OTS prosthesis was not able to reduce subsidence as it claimed.

## Background

Degenerative cervical spondylotic myelopathy (DCSM) is a debilitating condition characterized by compression of the spinal cord due to degenerated intervertebral discs or osteophytes, resulting in symptoms such as upper arm pain, numbness, muscle weakness, and impaired mobility. Consequently, this disorder significantly diminishes the patient's overall quality of life [1, 2]. The symptoms of DCSM can be significantly alleviated through surgical intervention involving neurological decompression and stability reconstruction. Among the various surgical approaches, anterior cervical corpectomy and fusion (ACCF) has gained widespread recognition since its initial description in the 1950s [3, 4]. However, the optimal implant for the post-decompression gap remains a subject of debate due to the persisting complications associated with current implants, such as subsidence or non-union [5–7]. Autologous iliac bone grafts and titanium mesh cages (TMC) have been extensively utilized in ACCF. However, the occurrence of postoperative iliac pain, infection, and the high subsidence rate of implants have prompted surgeons to explore novel implant alternatives [8–10]. With the rapid advancement of the Three-dimensional printing (3DP) technique [11–14], the 3DP trabecular structured prosthesis is purported to enhance bone ingrowth and mitigate stress shielding effects by incorporating appropriate trabecular spacing, thereby ensuring enhanced initial stability [15, 16]. Anterior cervical spinal surgery has emerged as a pioneering adopter of 3DP technology, encompassing both patient-specific (PS) and market-available “Off-The-Shelf” (OTS) implants [13, 14, 16–19]. Only a limited number of studies reported sporadic cases of PS 3DP implants with a short-term follow-up [20, 21]. Nevertheless, there is a paucity of clinical studies to verify the efficacy of 3DP OTS prosthesis in ACCF. This study aimed to find out if 3DP OTS prosthesis is superior to TMC in ACCF as it claimed.

## Methods

### Patient population and indications

Fifty-one patients with DCSM who received ACCF from January 2016 to January 2019 were enrolled in this study with an informed consent agreement. The inclusion criteria were: (1) patients with symptoms and signs of DCSM in which conservative treatment was ineffective; (2) Patients with single or two-level disc degeneration, where the herniated disc or osteophyte extends to the posterior margin of the vertebral body, or focal ligaments are thickened, resulting in spinal cord compression, ACDF is not expected to provide complete decompression. The exclusion criteria were: (1) Continuous ossification of the posterior longitudinal ligament (OPLL); (2) developmental spinal stenosis; (3) preexisting dysphagia; (4) cervical scoliosis or kyphosis; (5) history of rheumatoid arthritis; (6) history of cervical spine tumor; (7) history of allergy to materials used in the procedure; (8) history of cervical spine trauma with/without cervical spine surgery; (9) evidence of systemic or local infection; (10) Osteoporosis was diagnosed by DXA (T-score is less than -2.5). Patients were divided into two groups: The 3DP group consisted of 28 patients (16 males and 12 females), aged 53.2 ± 6.4 years, who underwent ACCF with a 3DP OTS prosthesis; The TMC group consisted of 23 patients (12 males and 11 females), aged 53.3 ± 6.5 years, who underwent ACCF with TMC. All patients were followed up for at least 24 months postoperatively. None of these patients were lost to follow-up.

### Surgical procedures

All surgeries were performed by the same senior surgeon (Kuang) using the standard Smith-Robinson method. The two intervertebral discs of the diseased segment are first removed, carefully removing the cartilage endplates and avoiding excessive damage to the endplates, followed by the removal of the middle vertebral body and removal of the posterior osteophytes with scrapers and Kerrison Rongeurs. After the decompression of the spinal cord and nerve roots, the cervical curvature was adjusted from a hyper-extended state to a physiological lordotic state. Subsequently, the Caspar retractor was released to maintain the gap at a similar height as the normal upper or lower segment. Lateral X-ray fluoroscopy was also used to check the height and position. In comparison with the adjacent normal vertebral space, there was no significant opening observed in the facet joint space, indicating an absence of excessive distraction [22]. The Casper retractor is also deployed upon implantation of the prosthesis to effectively restore the "natural height" of the segment to ensure congruity between the surgical space and implant dimensions. This reduces the occurrence of postoperative pain caused by over-extension and prevents rapid cage subsidence or loss of cervical curvature [23]. The width of the implant was determined by the distance between the two Luschka's joints. In the 3DP group, an appropriately sized OTS 3D-printed prosthesis (TITAN, AK Medical, Beijing, China) was implanted under fluoroscopy guidance. In the TMC group, titanium mesh cages (WEGO titanium mesh cage, WEGO, Weihai, China) were inserted with autogenous bone blended with porous bio-ceramic artificial bone (Dragonbio, Hubei, China) before implanted into the intervertebral space. The prostheses utilized in this study have diameter of either 12 or 14 mm. The upper and lower cover plates are absent in TMC. The inserted substitute, surrounded by the sharp ring of TMC, directly contacted with the vertebral endplates. In contrast, the exterior and interior of the 3DP prosthesis exhibit a porous trabecular structure with a flat surface on both ends without any sharp protrusion penetrating into the vertebral endplates. The same type of titanium anterior cervical plate (ATLANTIS, Medtronic Sofamor Danek, Memphis, USA) was attached and screw-locked to the front of the adjacent vertebrae of the surgical segment to stabilize the cervical spine. Drainage was placed after flushing and the wound was closed by layers.

### Outcome assessment

**Fig. 1:**
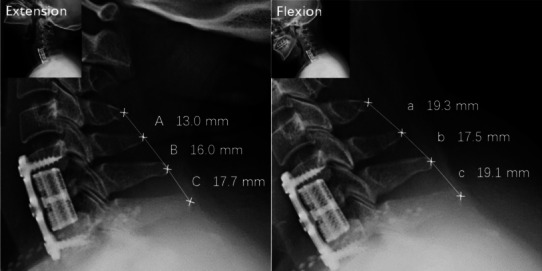
Measurement of interspinous movement (ISM) at superjacent level (C4-5) and operated levels (C5-7) on the 150% magnified flexion and extension radiographs. The superjacent ISM at C4-5 (A and a) was 6.3 mm, which indicated adequate dynamic motion (≥ 4 mm). ISM at C5-6 (B and b) and C6-7 (C and c) were 1.5 mm and 1.4 mm, which was inconsistent with the definition of fusion (< 4 mm)

**Fig. 2:**
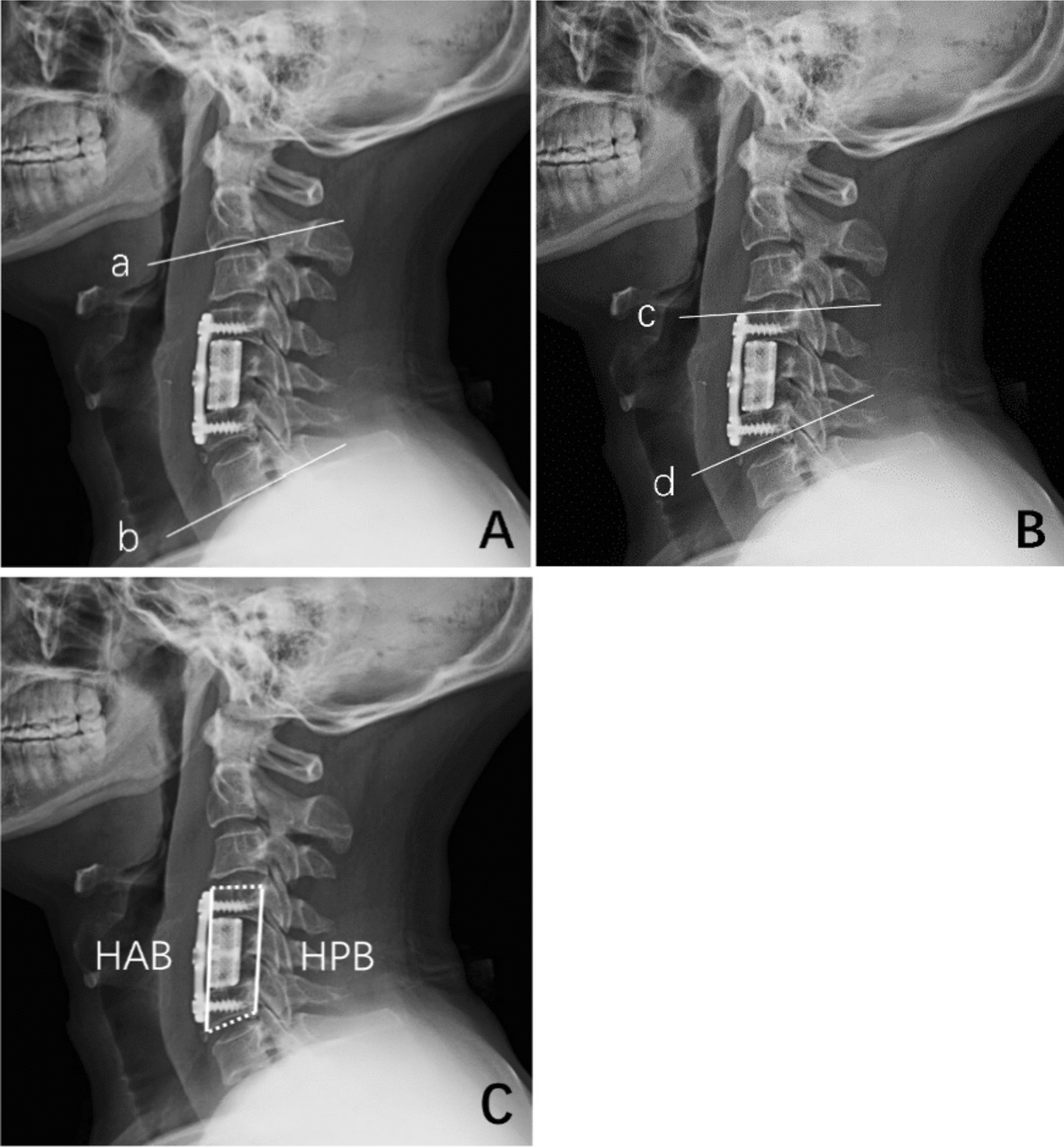
**A** The measurement method for cervical Cobb’s lordosis (CL): the angle formed by the upper end plate of C2 and lower end plate of C7 in neutral position (line a and line b); **B** the measurement method of the fused segment angle (FSA): the angle formed by the upper endplate of the superior vertebrae body and the lower endplate of the inferior vertebrae body in neutral position (line c and line d); **C** the measurement method for mean vertebral height (MVH): the mean value of the height of anterior (HAB) and the posterior border (HPB), The HAB and HPB were measured as the distance between the anterior and posterior points of the upper endplate of the superior vertebra and the lower endplate of the inferior vertebra

The hospital stays, operation time, and intraoperative blood loss were recorded. The Japanese Orthopedic Association (JOA) scores, and Neck Disability Index (NDI) were measured preoperatively and recorded at 1 day, 3, 6, 12, and 24 months postoperatively. The results of the procedures were evaluated by two independent investigators (Wang and Chen), and the procedures were graded according to Odom’s criteria. Disagreements were resolved by discussion and consensus with another independent expert (Y Li). The interspinous motion (ISM) < 1 mm and superjacent interspinous motion ≥ 4 mm confirm the fusion diagnosis on the 150% mag magnified flexion and extension radiographs [24] (Fig. 1). The cervical lordosis (CL) fused segmental angle (FSA) and the mean vertebral height (MVH) of the surgical segment were taken pre-operatively and at 1 day, 3, 6, 12, and 24 months post-operatively (Fig. 2). The MVH was defined as the average height of the anterior border (HAB) and height of the posterior border (HPB). Subsidence was defined as loss of more than 3 mm in any of the HAB and HPB measured heights compared with the 1 day after operation [25]. Two independent researchers evaluated the radiographs (Pan and Yuan), and the results of their measurements were analyzed by intra-class correlation coefficient for data consistency. Disagreements on fusion were discussed with another independent expert (Lü) and a consensus was reached to minimize the observer bias. The Chinese version of Short Form-36 (SF-36) was used to assess the health-related quality of life (HRQoL) of patients(X Li).

### Statistical analysis

Statistical analysis was performed using Statistical Product and Service Solutions 28.0 statistical software (SPSS Inc, Chicago, Illinois, USA). The mean and standard deviation (x ± S) were used to express the measurement data, and the count data was expressed by the number of cases and percentages. The comparison of measurement data between groups was performed by student’s t-test, and the Paired t-tests were used to compare differences in preoperative and postoperative measurement data within groups; the non-parametric test was used for comparison of count data, and the difference was statistically significant at *p* < 0.05. The consistency of data collected by different researchers was analyzed by intra-class correlation coefficient (ICC) (ICC < 0.4, poor consistency; ICC > 0.75, good consistency).

## Results

### Clinical outcomes

There were no statistically significant differences in general information of patients between the 3DP group and the TMC group (Table 1). There were no statistically significant differences observed in terms of operation time, blood loss, and hospital stays between the 3DP group and the TMC group (Table 2). A total of five cases of postoperative dysphagia were observed (2 cases in the 3DP group and 3 cases in the TMC group), and symptoms were alleviated one week later following symptomatic treatment. Both groups showed improvement in NDI and JOA scores after surgery (*p* < 0.05), but differences at each time point were not statistically significant (Table 3). The mental health status and social roles of the HRQoL scores in both groups were nearly equivalent to those of healthy individuals. Additionally, the SF-36 results also revealed there was no significant difference between the two groups in HRQoL (Table 4).

**Table 1:** General information of patients (mean ± SD)

Group	3DP group	TMC Group	*p*-value
Male/Female	16/12	12/11	0.365
Age (years)	53.2 ± 6.3	54.6 ± 5.7	0.426
Corpectomy segment			0.425
C3	4	3	
C4	5	4	
C5	10	8	
C6	9	8

**Table 2:** Clinical results of patients (mean ± SD)

Group	3DP group	TMC Group	*p*-value
Operation time (minutes)	85.2 ± 15.3	84.8 ± 15.6	0.929
Blood loss (ml)	133.4 ± 83.2	127.8 ± 81.0	0.815
Hospital stays (days)	7.3 ± 2.1	7.7 ± 1.7	0.422

**Table 3:** Preoperative and postoperative (NDI, JOA) (mean ± SD)

Group	3DP group	TMC Group	*p*-value
NDI			
pre-OP	24.3 ± 2.5	24.9 ± 3.0	0.199
1d P.O	20. 1 ± 2.4	20.6 ± 2.8	0.248
3 m P O	17.7 ± 2.0	17.5 ± 2.5	0.356
6 m P.O	15.5 ± 1.9	14.9 ± 1.9	0.140
12 m P.O	13.4 ± 1.7	13.0 ± 1.8	0.173
24 m P.O	11.7 ± 1.8	11.3 ± 2.0	0.244
JOA			
pre-OP	9.5 ± 1.4	9.3 ± 1.3	0.278
1d P.O	11.8 ± 1.3	11.5 ± 1.6	0.231
3 m P O	12.9 ± 1.2	12.7 ± 1.5	0.303
6 m P.O	13.8 ± 1.2	13.6 ± 1.5	0.289
12 m P.O	14.2 ± 1.1	14.0 ± 1.6	0.365
24 m P.O	14.6 ± 1.3	14.7 ± 1.5	0.410

*pre-OP* pre-operation, *1d P.O* 1 day post-operatively, *3 m P.O* 3 months post-operatively, *6 m P.O* 6 months post-operatively, *12 m P.O* 12 months post-operatively, *24 m P.O* 24 months post-operatively

**Table 4:** Results of the Short Form -(SF-)36 (Mean ± SD)

SF-36 dimensions	3DP group (*n* = 28)	TMC group (*n* = 23)	*p*-value
PF	46.8 ± 12.6	46.7 ± 2.4	0.950
RP	39.3 ± 7.6	37 ± 12.8	0.516
BP	51.4 ± 7.9	51.7 ± 8.9	0.893
SF	47.3 ± 5	43.5 ± 6.4	0.065
GH	44.3 ± 4	42.4 ± 4.5	0.167
VT	45.2 ± 18.2	44.3 ± 3.8	0.450
RE	55.9 ± 5.7	53.6 ± 16.6	0.639
MH	51.6 ± 3.9	49.9 ± 4.7	0.267
PCS	45.4 ± 7.5	44.5 ± 4	0.375
MCS	50 ± 5.9	47.8 ± 6.8	0.289
RCS	46.1 ± 5.9	43.4 ± 5.5	0.113

*PF* physical functioning, *RP* role limitations due to physical problems, *BP* bodily pain, *SF* social functioning, *GH* general health perceptions, *VT* vitality, *RE* role limitations due to emotional problems, *MH* mental health, *PCS* physical component summary, *MCS* mental component summary, *RCS* role-social component summary

### Radiological outcomes

**Fig. 3:**
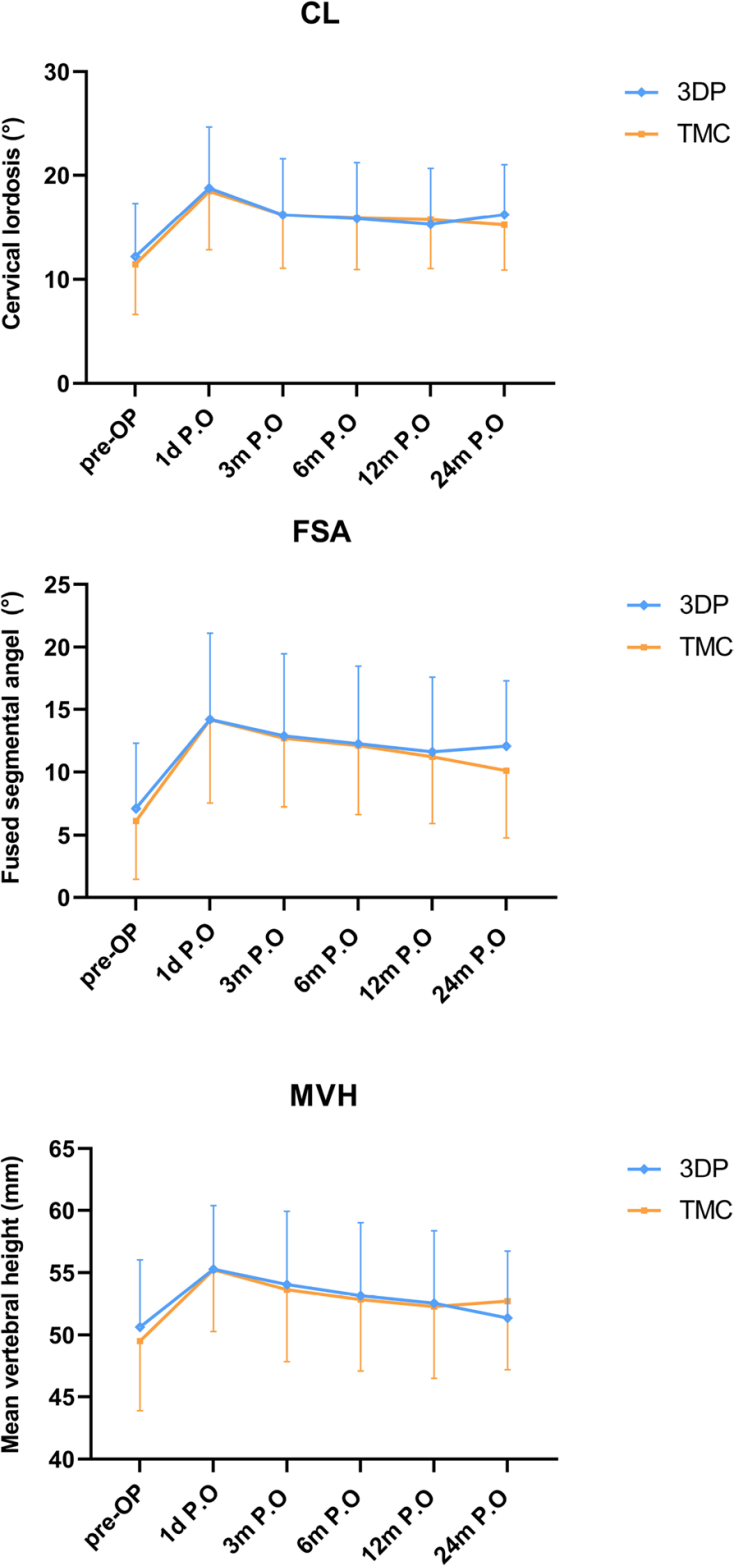
The radiological outcomes. There was no statistically significant difference between the two groups at each follow-up time point. *pre-OP* pre-operation, *1d P.O* 1 day post-operatively, *3 m P.O* 3 months post-operatively, *6 m P.O* 6 months post-operatively, *12 m P.O* 12 months post-operatively, *24 m P.O* 24 months post-operatively

The radiological data have good consistency derived from measurements by Wang and He. No instances of screw loosening or plate breakage were observed in either group, and successful bone fusion was achieved in all cases except for one patient within the 3DP group. The incidence of subsidence in the 3DP group at 3, 6, 12, and 24 months postoperatively was as follows: 7 cases (25.0%), 9 cases (32.1%), 10 cases (35.7%), and 10 cases (35.7%) respectively; while in the TMC group, it was observed as: 6 cases (26.1%), 7 cases (30.4%), 8 cases (34.8%), and 8 cases (34.8%). The observed differences in subsidence rate between the two groups did not reach statistical significance (*p* >0.05). The radiological parameters showed improvement at the last follow-up compared to the pre-operative measurements in each group (*p* <0.05) (Table 5). The radiological parameters did not exhibit statistically significant differences between the two groups at each follow-up time point. (*p* >0.05) (Fig. 3). The results showed that the intraobserver ICCs for CL, FSAs, and MVH were 0.990, 0.982, and 0.984, respectively, while the interobserver ICCs were 0.976, 0.963, and 0.968, respectively.

**Table 5:** Preoperative and postoperative radiological data (CL, FSA, MVH) (mean ± SD)

Group	3DP group	TMC Group	*p*-value
CL (°)			
pre-OP	12.2 ± 5.1	11.4 ± 4.9	0.599
24 m P.O	16.2 ± 4.8	15.2 ± 4.3	0.472
FSA (°)			
pre-OP	7.1 ± 5. 2	6.1 ± 4.7	0.494
24 m P.O	12.1 ± 5.2	10.1 ± 5.4	0.197
MVH (mm)			
pre-OP	50.6 ± 5.4	49.5 ± 5.6	0.481
24 m P.O	51.3 ± 5.4	52.7 ± 5.5	0.391

*pre-OP* pre-operation, *24 m P.O* 24 months post-operatively, *CL* cervical lordosis, *FSA* fused segmental angel, *MVH* mean vertebral height

### Costs

The costs of both groups were shown in Table 6. The total cost was higher in the 3DP group (*p* < 0.05). The cost of the prosthesis was higher in the 3DP group (*p* < 0.05). Differences in internal fixation and other costs between the two groups were not statistically significant (*p* > 0.05).

**Table 6:** Hospitalization cost of the patients

	3DP group (*n* = 28)	TMC group (*n* = 23)	*p*-value
Cost (thousand US dollars)	15.08 ± 1.3	14.0 ± 0.9	0.001
Prosthesis (with or without bone graft)	3.29 ± 0^a^	1.12 ± 0^a^	0.001
Internal fixation	0.54 ± 0.1	0.50 ± 0.1	0.083
Other costs	11.48 ± 1.3	11.1 ± 0.9	0.238

^a^The cost of the TMC group included both TMC and the bone graft, while the one of the 3DP group did not contained bone graft

## Discussion

The anterior cervical corpectomy and fusion achieve satisfactory decompression efficiency in the treatment of DCSM by relieving the compression of the spinal cord directly [1, 2]. It not only provides preliminary stability of the cervical spine but also plays a significant role in the maintenance of cervical lordosis. However, concerns have arisen regarding complications associated with the implant, including subsidence, non-union, screw migration, or implant breakage, which can ultimately lead to fixation failure [5–7]. Spine surgeons have been seeking implants with fewer implant-related complications [5–10]. The titanium mesh cage has replaced the autologous iliac crest bone for its initial stability and satisfactory fusion rate while avoiding harvest-related infection and injury, which facilitates pain relief and patient recovery [10]. However, most implants that are made of titanium alloy (Ti-6Al-4 V) have a high radiological opacity, which makes it difficult to evaluate their fusion on CT. Thus, fusion must be evaluated by observing the angle changes of the fused segment on the lateral flexion–extension radiograph. Besides, a high risk of subsidence of the titanium mesh cage has been reported due to its high stiffness [9]. Riew et al. evaluated the accuracy of four radiographic fusion criteria for determining anterior cervical fusion status [24]. These criteria include interspinous motion (ISM) < 1 mm with superjacent ISM ≥ 4 mm on dynamic radiographs [26], conventional bridging bone on CT scans [27], extra-graft bridging bone (ExGBB) and intra-graft bridging bone (InGBB) observed on multi-directional reconstructed CT scans [28]. He found that the ExGBB has the highest accuracy, sensitivity, and specificity compared to the other criteria, but the ISM criterion also demonstrated a similar accuracy to that of the conventional bridging bone criteria on CT scans. Despite the advantages of CT scans in detecting bridging bone and arthrodesis, the ISM criteria have advantages over CT scan in terms of cost and radiation exposure and also showed high inter- and intra-observer reliability and accuracy, as suggested in the article, making it the diagnostic standard for our patients.

The development of 3DP technology has led to the advancement of 3DP prostheses [11–13]. The 3DP prosthesis, characterized by its exquisite microstructure and excellent mechanical properties, has been developed with the aim of reducing complications associated with implants and shortening surgical duration [13]. The 3DP prosthesis currently used are divided into patient-specific (PS) and market-available “Off-The-Shelf” (OTS) ones according to the design requirements [13, 16–19]. The PS prosthesis is designed using computer-aided design (CAD) software, utilizing anatomical data obtained from the patient's CT scans. This CAD modeling technique ensures precise reconstruction of the patient's unique anatomy and enables the fabrication of a customized prosthesis tailored to meet specific surgical requirements and anticipated intraoperative needs. The clinical efficacy and radiological results of PS 3DP prosthesis used in single-segment ACCF were reported sporadically. Amelot et al. used PS 3DP prosthesis for vertebral reconstruction in ACCF and achieved satisfactory results [20]. Li et al. [21] and Lu et al. [29] compared the PS 3DP prosthesis with the titanium mesh cage (TMC) and concluded that PS-3DP prosthesis achieved comparable results in cervical lordosis reduction, neurological function, and HRQoL score, and the subsidence rate. Compared to the OTS implants, PS implants not only required less operative time but also exhibited reduced subsidence rates, potentially attributed to their enhanced geometric consistency achieved through mimicking the surface morphology of the endplate [30–33]. However, the extended production time and high manufacturing costs impose limitations on its practical application. Consequently, a 3DP OTS prosthesis was developed based on anatomical data from a large population, offering the potential to replace PS 3DP implants in most spine surgeries due to reduced costs and production time. Nevertheless, there is a scarcity of studies comparing clinical and radiological outcomes between the 3DP OTS prosthesis and commonly used TMCs, leaving the efficacy of the 3DP OTS prosthesis unknown [34]. In contrast to the PS 3DP prosthesis, our study found that the 3DP OTS prosthesis did not exhibit superior clinical or radiological results compared to conventional TMC.

Subsidence was defined as the migration of the intervertebral fusion device into the adjacent vertebral body, caused by stress concentration on the contact surface between the implant and the bone, which leads to implant failure, as well as undesirable consequences such as non-fusion and cervical kyphosis [35, 36]. Several risk factors may be associated with graft subsidence, including graft-related factors such as material composition, structure, shape, size, and dimension. Currently, both the 3DP OTS prosthesis and TMC are made of titanium alloy (Ti6Al-4 V) for their load-carrying capacity, cellular adhesion, and corrosion resistance [37, 38]. The stress shielding effect resulting from the mismatch in elastic modulus between titanium alloy and bone tissue induces higher local stresses at the edges, leading to bone resorption, non-union, and subsidence [39–42]. In our study, a total of 18 cases (35.3%) of subsidence were observed, with 10 cases (35.7%) in the 3DP group and 8 cases (38.1%) in the TMC group, respectively. However, no statistically significant difference was found between the two groups. Therefore, it can be concluded that the use of 3DP OTS prosthesis does not demonstrate superiority over TMC in preventing subsidence in ACCF.

Previous researches have demonstrated a significant correlation between implant shape, size, and dimensions and the occurrence of subsidence [43–45]. Furderer et al. [46] said that rectangular implants have a better-carrying capacity and could reduce the occurrence of subsidence. The reduction in contact area has been demonstrated by numerous biomechanical studies to result in stress concentration, which is implicated in the development of subsidence [43, 44, 46–49]. A study reported that the implementation of porous structures in 3DP prosthesis facilitates improved stress distribution, mitigates stress shielding effects, and reduces subsidence [50]. Some researchers compared the mechanical performance of individual PS designs with generic OTS designs and reported that the geometric conformity of the bone-implant interface of PS designs minimizes point-loading and resulting stress rise through a more uniform load distribution [34, 51]. The design of the OTS prosthesis failed to achieve similar geometric conformity as the PS ones. This may result in stress concentration, which may contribute to the result in our study that the subsidence rate of the 3DP OTS prosthesis was not superior to TMC.

Another reason is that although the 3D-printed prosthesis mimics the structure of bone trabeculae to facilitate bone ingrowth, it does not stimulate osteogenesis due to the absence of graft bone. During the fusion process between the implant and the vertebral body, it has been reported that osseointegration at the cortical and cancellous sites of long bones does not directly translate into intervertebral fusion because the endplate is a laminar porous structure composed of fused bone trabeculae, and fusion of the intervertebral fusion device to the endplate is similar to osseointegration at cancellous sites, even when implanted in a press-fit fashion. Consequently, the fusion between the implant and the vertebral body became non-robust, and subsidence occurred naturally. This may also be the reason for the non-fusion case in the 3DP group although her age was only 32 years and had no history of smoking, drinking, osteoporosis, obesity, or other comorbidity.

The bone mineral density (BMD) plays an important role in preventing subsidence [52–55]. The decreases in BMD aggravate the degree of elastic modulus mismatch between the vertebral body and the implant. Smoking, age, and sex may be involved in the occurrence of subsidence by affecting bone healing and BMD [52, 55, 56]. In our study, we excluded patients with osteoporosis to ensure that there would be no differences in bone quality that could affect the results.

The vertebral bone quality is a major factor affecting fixation. In terms of BMD assessment, dual-energy X-ray absorptiometry (DEXA) is considered the “gold standard” due to its simplicity and cost-effectiveness, with low-level radiation exposure. The t-value of DEXA less than 2.5 was used to diagnose osteoporosis. Although DEXA is evidently effective, there exist several methodological constraints in accurately quantifying BMD in patients with degenerative spine disorders. The presence of osteophytes, articular hypertrophy, and soft tissue deterioration, such as abdominal vascular wall calcification, can affect the lumbar BMD value and cause it to be exaggerated [57]. Besides, the measurement sites of DEXA are the lumbar spine and femur, not the cervical spine. It is still unclear whether there is a correlation between the actual value of the cervical spine and the lumbar spine, and how much bias there is. Therefore, there may be no overall osteoporosis but low bone mass density locally. Therefore, this study may not be able to completely rule out osteoporosis in the cervical spine. Recently, BMD assessment using computed tomography (CT) Hounsfield units (HU) has been developed as a modern trustworthy approach to measuring bone quality. HU values have been found to be favorably correlated with both vertebral compressive strength and DEXA-measured BMD [58]. Although the HU values offer the advantage of self-selected area measurement, there remains a lack of established diagnostic standards for osteoporosis, particularly in localized regions.

The preservation of the vertebral endplate and avoidance of intervertebral over-distraction have been demonstrated in studies to possess superior biomechanical properties, effectively preventing bone graft collapse, reducing kyphotic deformity, and maintaining intervertebral space height [48, 55, 59–62]. The endplate is a laminar porous structure composed of fused bone trabeculae. It contains pore-like forms of varying sizes, more in the central region and less in the periphery, which are the anatomical basis for the nutritional supply of the intervertebral disc and the maintenance of stress stability in the vertebral body. Therefore, the location of the implant also has an impact on the subsidence. The graft should be placed in the anterior periphery position of the endplate [60]. In this study, both groups of cases were operated by the same physician, who consistently followed identical procedures for implant insertion and end plate disposition in both groups. Consequently, potential surgical errors resulting from different surgeons were effectively eliminated.

Additionally, it has been postulated by certain researchers that the extent of corpectomy may serve as a risk factor for severe subsidence [33], with a higher likelihood of subsidence occurring in the inferior vertebrae, potentially attributed to the biomechanical forces acting upon them.

The maintenance of cervical lordosis is associated with subsidence, as previous studies have suggested that implant subsidence may contribute to the development of kyphosis [63]. Li et al. reported that a larger T1 Slope may be associated with subsidence [48]. An et al. also believed that more subsidence was observed when the T1 slope was larger, and the CL was corrected markedly [64]. The increase of CL and the large obliquity of the T1 slope were regarded as an inducer of subsidence. In our study, although both groups of patients demonstrated improvement in CL compared to their pre-operative status, there was no statistically significant difference observed between the two groups at each follow-up time point. This lack of significance may be attributed to the non-statistically significant difference in subsidence rate between the two groups.

The association between sagittal parameters and surgical outcomes extends well beyond that. The NDI score serves as a pivotal indicator for evaluating clinical outcomes. Rao et al. discovered that the mismatch between T1 slope and C2-C7 lordosis (T1s-CL) was associated with a more unfavorable postoperative NDI score compared to the matched group in laminoplasty studies [65]. Similarly, Lan et al. emphasized the significance of C2-7 SVA as a pivotal parameter for predicting the surgical outcome in patients with cervical kyphosis in their study [66].

Although both groups achieved comparable clinical and radiological outcomes in our study, the 3DP group exhibited a higher cost profile, with greater total hospital expenses compared to the TMC group. To be specific, the cost of internal fixation and the other costs of the two groups were similar. However, the average prosthesis cost of 3DP was nearly 3 times of TMC, even including bone graft. China spent USD 298 per person on health care and the per capita disposable income was USD 5,189 in 2022. The difference between the graft cost of 3DP and TMC is roughly 40% of annual per capita income (USD 2170 vs. USD 5189). Therefore, cost-effectiveness should be carefully considered in the selection of implants.

Our study had several limitations, including its single-center retrospective design with a relatively small sample size. To enhance the persuasiveness and confirm these findings, it is crucial to conduct a randomized multi-center prospective study with larger sample size. Furthermore, our study did not elucidate the specific relationship between local BMD, smoking, obesity, other comorbidities, and subsidence. Additionally, we excluded only patients with osteoporosis and did not compare bone mass between the 3DP group and the TMC group. Lastly, further research is needed to investigate the long-term effect of subsidence on post-operative cervical sagittal balance as well as its association with symptoms.

## Conclusions

In the treatment of single-segment DCSM with ACCF, both 3DP OTS prosthesis and TMC achieved satisfactory outcomes, yet no statistically significant differences were observed between the two implants in terms of clinical and radiological results. The purported claim of reducing implant subsidence by the 3DP OTS prosthesis was not substantiated. However, it is worth noting that the 3DP OTS prosthesis incurred higher costs compared to TMC.

## Data Availability

The datasets used and/or analysed during the current study are available from the corresponding author upon reasonable request.
